# Fluorescence Characterization of Clinically-Important Bacteria

**DOI:** 10.1371/journal.pone.0075270

**Published:** 2013-09-30

**Authors:** Lewis R. Dartnell, Tom A. Roberts, Ginny Moore, John M. Ward, Jan-Peter Muller

**Affiliations:** 1 UCL Institute for Origins, University College London, London, United Kingdom; 2 The Centre for Planetary Sciences at UCL/Birkbeck, University College London, London, United Kingdom; 3 Centre for Mathematics and Physics in the Life Sciences and Experimental Biology (CoMPLEX), University College London, London, United Kingdom; 4 Clinical Microbiology & Virology, University College London Hospitals NHS Foundation Trust, London, United Kingdom; 5 The Advanced Centre for Biochemical Engineering, University College London, London, United Kingdom; 6 Mullard Space Science Laboratory, University College London, Holmbury St. Mary, Surrey, United Kingdom; University of California, Irvine, United States of America

## Abstract

Healthcare-associated infections (HCAI/HAI) represent a substantial threat to patient health during hospitalization and incur billions of dollars additional cost for subsequent treatment. One promising method for the detection of bacterial contamination in a clinical setting before an HAI outbreak occurs is to exploit native fluorescence of cellular molecules for a hand-held, rapid-sweep surveillance instrument. Previous studies have shown fluorescence-based detection to be sensitive and effective for food-borne and environmental microorganisms, and even to be able to distinguish between cell types, but this powerful technique has not yet been deployed on the macroscale for the primary surveillance of contamination in healthcare facilities to prevent HAI. Here we report experimental data for the specification and design of such a fluorescence-based detection instrument. We have characterized the complete fluorescence response of eleven clinically-relevant bacteria by generating excitation-emission matrices (EEMs) over broad wavelength ranges. Furthermore, a number of surfaces and items of equipment commonly present on a ward, and potentially responsible for pathogen transfer, have been analyzed for potential issues of background fluorescence masking the signal from contaminant bacteria. These include bedside handrails, nurse call button, blood pressure cuff and ward computer keyboard, as well as disinfectant cleaning products and microfiber cloth. All examined bacterial strains exhibited a distinctive double-peak fluorescence feature associated with tryptophan with no other cellular fluorophore detected. Thus, this fluorescence survey found that an emission peak of 340nm, from an excitation source at 280nm, was the cellular fluorescence signal to target for detection of bacterial contamination. The majority of materials analysed offer a spectral window through which bacterial contamination could indeed be detected. A few instances were found of potential problems of background fluorescence masking that of bacteria, but in the case of the microfiber cleaning cloth, imaging techniques could morphologically distinguish between stray strands and bacterial contamination.

## Introduction

### Healthcare-associated infections

Healthcare-associated infections (HCAI or HAI), or nosocomial infections, are those acquired by a patient during the period of hospitalization itself, and present a serious threat to health, especially to patients already with weakened immune systems or recovering in intensive care wards.

In the United States, there are an estimated 2 million cases of HAI every year, of which around a quarter occur in intensive care units and are estimated to cause 90,000 deaths a year [1]. The Centers for Disease Control and Prevention estimates that 5% to 10% of hospital patients develop a HAI, and they are one of the top ten causes of death in the United States. HAI in the US cost an estimated $4.5 billion to $6.5 billion extra per year in patient treatment [2]. In the UK, 7.6% of acute hospital patients acquire an HAI, around a sixth of which are caused by methicillin-resistant *Staphylococcus aureus* (MRSA) and another sixth by *Clostridium difficile* [3]. National, evidence-based guidelines were developed over 1998-2000 by the Department of Health to help prevent HAI in National Health Service hospitals in the UK [4], and significant reductions in health-care acquired *Clostridium difficile* and MRSA infections in 2010-2011, continuing a year-on-year trend, were reported by the national surveillance programme [5]. But HAIs remain a substantial problem in patient care and there is much room for improvement in procedures for limiting transmission between patients and healthcare professionals, or instrumentation able to detect contamination before infection can occur.

Approximately 20-40% of HAI are due to cross-infection between patients by the hands of health care personnel, either via by direct contact with infected patients or touching contaminated clinical surfaces: environmental reservoirs. Such environmental contamination is particularly important for transmission of methicillin-resistant *Staphylococcus aureus*, vancomycin-resistant *Enterococcus* spp., *Clostridium difficile*, and *Acinetobacter* spp. [6]. For example, Layton et al. (1993) [7] find that outbreaks of MRSA were caused by a contaminated blood pressure cuff, and Manian et al. (1996) [8] also implicate contaminated blood-pressure cuffs in the outbreaks of *Clostridium difficile* in their medical centre, with over half occurring in the intensive care units.

Currently, hospitals are not able to routinely check for contamination due to limited resources. However, if there has been an outbreak, the hospital is likely to test the environment by swabbing for the presence of pathogenic bacteria, which requires a wait whilst the culture grows. There is therefore a demand for novel surveillance instrumentation that can detect microbial contamination *before* it causes HAI. Decreasing environmental contamination may help control transmission of HAIs [9], and developing technologies to assist in the surveillance and detection of contamination could therefore make major contributions.

One promising technique that could prove effective in detecting the presence of bacteria cells where they ought not be, such as on supposedly-cleaned surfaces in an intensive care ward or surgical theatre, is fluorescence. A fluorescence-based detection device would provide a surveillance function to confirm effective routine cleansing and disinfection of hospital surfaces, the proper decontamination of reusable medical devices or detect contamination of single-use equipment such as central lines. For example, an HAI outbreak of *Pseudomonas aeruginosa* reported in Germany was spread due to an inappropriate cleaning solution used by hospital staff [10], which may have been preventable by a contamination surveillance instrument. *Pseudomonas aeruginosa* is an opportunistic pathogen of humans, and excretes an extracellular siderophore compound, pyoverdine, to scavenge iron from its environment that is itself fluorescent [11].

### Fluorescence-based detection and identification

Fluorescence-based detection systems have already proved themselves to be sensitive and discriminatory, and have been used in a number of similar applications to discern trace amounts of different targets in diverse contexts.

Fluorescence techniques are well developed in flow cytometry, a tool in cell biology for rapid analysis of large populations of cells as they pass in single file between laser source and photodetectors. Flow cytometry utilises either the intrinsic fluorescence (autofluorescence) of cellular molecules [12] or the addition of extrinsic fluorescent stains or antibody probes [13,14], and is now being increasingly employed for routine analyses in environmental or industrial microbiology [15].

Other prior applications of fluorescence techniques include assessing the presence of organic compounds or pollutants in the environment [16–18], identifying potentially pathogenic or toxic microorganisms in environmental water or food preparation [19–22], and detecting trace concentrations of microorganisms in glacial [23] and Antarctic ice [24], Antarctic sandstone [25], and the Atacama desert [26].

Beyond detection of small numbers of contaminant or environmental microbes, fluorescence-based analytical techniques have progressed to the stage of additionally identifying the organisms present. Ammor (2007) [27] provides a recent review of the applications of autofluorescence for the detection and identification of bacteria, and demonstrates that the technique is simple (does not require the application of labels), fast, and reliable. For example, fluorescence can successfully characterize marine and freshwater plankton, including those responsible for toxic algal blooms [22,28–31]. More specifically within medicine, fluorescence characterisation has provided insights into the development and progression of cancerous tissue [32–34]. The fluorescence of extracellular pyoverdines has been used to distinguish between cultures of certain strains of *Pseudomonas* [35,36]. Leblanc and Dufour (2002) [37] successfully distinguished between different species of bacteria using principal component analysis (PCA) of the autofluorecence of the aromatic amino acid, nucleotide and NADH components of the cell (thus without needing the addition of fluorescent stains or probes). Using a similar technique, it has been shown that lactic acid bacteria (*Lactobacillus* sp.) and yeast (*Saccharomyces* sp.) colonising meat products can be distinguished [38,39]; Bhartia et al. (2008) [40] differentiate classes of organic molecules, cells and spores; Sohn et al. (2009) [21] could distinguish between the three most commonly identified commensal and pathogenic bacteria in food: *Escherichia coli*, *Salmonella* spp, and *Campylobacter* spp.; and Giana et al. (2003) [41] successfully discriminated between the clinically important *Escherichia coli*, *Enterococcus faecalis* and *Staphylococcus aureus*.

Whilst encouraging for the potential offered by fluorescence-based techniques, many of these studies were performed under idealised laboratory conditions, with pure cultures of limited numbers of model organisms and applying statistical analyses to the extensive datasets produced by large lab-bench spectrofluorimeters. Fluorescence has also been utilised in attempts to analyse the composition of aerosols, such as allegen-containing droplets [42] or in remote sensing instruments employing high power lasers at 266 or 355 nm and telescopic optics trialled for the stand-off detection of aerosolised bacteria in a biological warfare attack [43]. Whilst such systems can very sensitively detect emitted fluoresce, the challenge is in discriminating between, for example, bioweapon microorganisms and innocuous strains, or other environmental fluorophores, particularly with only one or two excitation wavelengths. Estes et al. (2003) [44] address this classification problem using a pulsed xenon lamp and three filters to provide different excitation wavelengths for detecting microbial contamination in the food industry.

Fluorescence-based detection and even characterisation has been demonstrated for environmental or food-contaminant microbes, no instrumentation has yet been developed for bacterial detection in a clinical setting at the macroscale using standoff, ‘remote sensing’, detection. The research reported here represents the initial key stage of experimental work required for the specification and design of just such a device in the interests of combating HAI. In certain clinical environments with exacting cleanliness standards, such as surgical theatres or high dependency wards, where patients have an increased vulnerability to HAIs, the problem is more constrained and thus tractable than environmental applications. Within such supposedly clean environments the detection of any bacteria or other contamination indicates an improper cleaning protocol, which can then be repeated before any contaminants have the opportunity to trigger an HAI outbreak. It is necessary to first quantify the full fluorescent response of many clinically-relevant bacteria in order to identify the most promising fluorescent features, and thus the excitation and emission wavelengths to target.

### Characterisation of fluorescent response

A full characterisation of the fluorescent response of a target, detailing the fluorescence intensity as a function of both excitation and emission wavelength, is provided by an Excitation-Emission Matrix (EEM). An EEM is generated by recording multiple emission spectra from the target as the excitation wavelength is incremented across a broad range. This stack of emission spectra as a function of excitation wavelength constitutes a three-dimensional datacube: a matrix of excitation–emission–intensity points. A meaningful way of visually representing this information-rich EEM datacube is as a two dimensional map of colour-coded emission intensity, plotted with contour lines of iso-intensity. Fluorescent features appear in this representation as peaks located at specific coordinates in the excitation–emission parameter space. Due to scattered excitation light, EEMs exhibit a diagonal ridge of high intensity with equation y=x, and a similar intense line with equation 2y=x from the first harmonic of the scattered excitation light permitted through the spectrometer diffraction grating. Only emission measured from the sample within these boundaries on the EEM is relevant, and these excluded regions have been blanked out in all EEMs displayed here. It should be noted that these laboratory spectrofluorimeters are not radiometrically calibrated and so cannot report absolute emission intensities. Consequently, it is not possible to assess sensitivities or detection thresholds from existing commercial devices; such work requires the construction of a prototype of the proposed surveillance device.

### Objectives

There is a need for a hand-held device to detect contaminant microbes in improperly cleaned clinical surfaces and equipment in rapid sweeps of a hospital environment, highlighting regions that require proper cleaning before a potential HAI outbreak. Such a device would be most readily applicable to clinical areas demanding the highest cleanliness standards, such as high dependency wards or surgical theatres, but may also prove effective in general wards with greater levels of background noise. To be appropriate for widespread use throughout health services, a device must be inexpensive, readily portable, and simple-to-use without specialist training. Such an instrument may thus be limited to one (or at most a few) excitation wavelength and a suitably filtered imaging system. There are therefore two clear objectives for this research, both of which must be resolved for the specification of a hand-held fluorescence-based surveillance device.

First, to characterise the complete fluorescence response – by constructing excitation-emission matrices (EEMs) over a broad range of wavelengths – of a wide variety of clinically-important bacterial strains in order to determine potential fluorescence features and the optimal excitation and emission wavelengths to target.

Second, to similarly generate EEMs of common hospital cleaning products and clinical surfaces to assess the potential issue of background noise masking bacterial contaminants.

To this end, we generated high-spectral-resolution EEMs from multiple samples of four Gram-positive bacteria: *Staphylococcus aureus*, *Staphylococcus carnosus*, *Clostridium difficile* strain CD630, *Clostridium difficile* strain R20291 and five Gram-negative bacteria: *Klebsiella pneumoniae*, *Serratia marcesens*, *Proteus mirabalis*, *Pseudomonas aeruginosa*, and *Escherichia coli*. *Staphylococcus carnosus* is more associated with contamination in the food industry, rather than clinical occurrence like *S. aureus* [45], but remains of interest and so is considered here alongside pathogenic *S. aureus*. We have also analysed multiple sites on 10 different representative clinical surfaces: microfiber cleaning cloth (Johnson-Diversey UK Ltd, Northampton, UK), ward computer keyboard, blood pressure cuff, nurse call button, and six different bed-rail materials, as well as two biocidal cleaning products: Actichlor (chlorine releasing agent) and Tristel Fuse (chlorine dioxide-based product).

## Method

### Bacterial culturing conditions

Nine bacterial strains were cultured for analysis, as listed above; all selected for their importance as potential clinical contaminants causing HAI. All except the *C. difficile* strains were cultured in nutrient broth in conical flasks with constant agitation at 180 rpm to late log-phase of growth. The two *C. difficile* strains were grown in brain heart infusion (BHI) broth in tissue culture flasks in an anaerobic chamber at the Eastman Dental Institute, London (in collaboration with Haitham Hussain). All bacterial strains were incubated at 37°C, except for *Serratia marcesens* which was grown at 30°C.

Regular sterile sampling (roughly every hour) from the growth cultures and measurement of the optical density at 600 nm (OD_600_), as well as dilution series and colony forming unit (CFU) assays, tracked the population density throughout the growth curve. This allowed the accurate determination of the exponential phase of growth, and all liquid cultures were sampled for fluorescence analysis at three or four evenly spaced points (every two hours) between the early- and late-log phase of growth, so as to minimise time-of-culture effects on inter-sample variability.

Due to the high concentration of organic molecules in the growth media, the bacteria need to be washed of their culture media in order to measure the fluorescence of the cells themselves. Thus, prior to analysis, all experimental samples of microorganisms were washed twice by centrifuging at 9000 g for 10 minutes and resuspending the bacterial pellet in an equal volume of Phosphate Buffer Solution (PBS). PBS contains negligible levels of organic carbon and so does not interfere with the measured fluorescence of the cell suspension (see 46). A single emission spectrum was obtained for the cell sample from excitation at 280 nm to check that the tryptophan fluorescence signal wasn’t saturating, with the cell suspension then diluted as necessary to ensure this before the full excitation-emission matrix data was acquired.

In addition, a liquid culture of *Pseudomonas aeruginosa* was grown in a minimal medium consisting of M9 salts, Ca/Mg salts, glucose, L-serine and trace elements, with 1% iron chloride (FeCl_3_). This was necessary in order to obtain spectral data on pyoverdine, an iron-chelating compound excreted by the organism into the environment. This siderophore compound is highly fluorescent, and so may offer a promising target for detection instrumentation, but as it is produced extracellularly would be lost before analysis by the above medium-washing protocol.

### Fluorescence measurements

The fluorescent emission of all samples was measured with a PerkinElmer LS55 Fluorescence Spectrometer (PerkinElmer, Cambridge, UK). This instrument consists of a pulsed xenon arc lamp light source, excitation and emission scanning monochromators, a sample compartment with a cuvette holder, and a photomultiplier tube (PMT) detector. Slightly different protocols are used for liquid samples or solid surface samples, as explained below. As stated before, the input and output of such a device cannot be radiometrically calibrated.

#### Liquid samples

For the bacterial cultures, 3 ml of the cleaned cell samples was pipetted into a 1 cm UV-C quartz cuvette (Fisher, Loughborough, UK) and placed in the sample holder. Before each new sample, the cuvette was swilled with 70% ethanol solution and then drained, and the outer walls wiped with the ethanol solution to remove contaminants, including fingerprints. Cell samples were diluted in PBS and an initial emission spectrum recorded at an excitation of 220 nm to check the peak height of tryptophan fluorescence. This dilution-test process was reiterated for each sample until the tryptophan signal was strong but not saturating the detector. Thus, the samples were scanned for a full excitation-emission matrix at densities of between 1x10^7^ and 1x 10^8^ cells/ml, dependent on the sample.

Data for a full excitation-emission matrix was collected as follows. The spectrometer was programmed to sweep through an excitation range between 200 nm and 800 nm, with an increment of 10 nm, thus yielding a total of 61 emission spectra for each sample. At every excitation wavelength, an emission spectrum was recorded between 200 nm and 900 nm, using an excitation slit width of 8.0 nm, and an emission slit of 5.0 nm, with data points logged every 0.5 nm at a scan speed of 1000 nm/min. The complete fluorescence data set collected for each sample is thus a three-dimensional datacube, composed of emission intensity measurements at over 85,000 excitation–emission wavelength combinations. An EEM was created for each of the three or four samples of the bacterial culture taken during its exponential growth phase, and averaged to improve the signal-to-noise ratio.

In addition, liquid samples of the cleaning fluids typically used on a hospital ward were analysed. These were Actichlor and Tristel Fuse. The Actichlor disinfectant tablets were dissolved in one litre of reverse-osmosis water as directed to make a 1000 ppm available chlorine solution. The Tristel Fuse disinfectant was mixed as directed on the packet.

#### Solid surface samples

**Figure 1:**
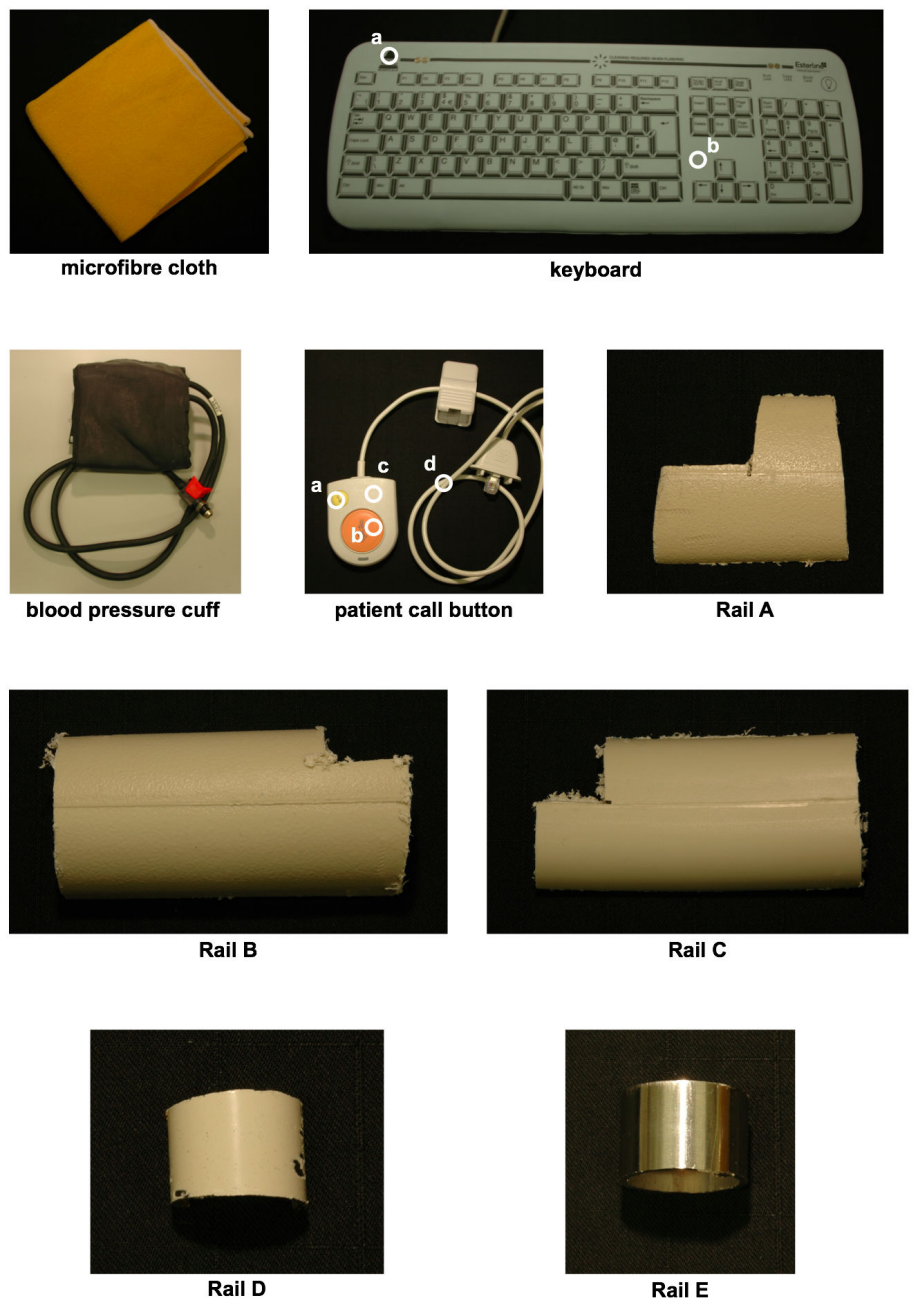
Photographs of solid surfaces typical of a hospital ward analysed for background fluorescence. Some of the samples have a heterogeneous composition and so different representative regions were analysed. These are labelled as follows: keyboard, a. black text, b. white background; patient’s nurse call button, a. yellow button, b. orange button, c. white casing, d. wire.

A number of pieces of equipment and sample surfaces typically found in a clinical setting were provided by Dr Ginny Moore (Clinical Microbiology and Virology, University College London Hospitals NHS Foundation Trust, London) for analysis. These were: microfiber cleaning cloth, silicone cover for ward computer keyboard, a blood pressure cuff, a patient’s bed-side nurse call button. Five different samples of bed rails commonly found within a ward were also analysed: Rail A (polypropylene with polyester powder coating), Rail B and Rail C (both polypropylene), Rail D (nylon polyester painted mild steel), Rail E (stainless steel), as shown in Figure 1. Surface samples were wiped clean with ethanol before analysis.

For the solid surface samples, the PerkinElmer fibre-optic cable attachment to the spectrometer was used. This accessory replaces the cuvette holder in the front bay of the instrument, and carries excitation light along one-half of a roughly one metre-long fibre optic bundle. Emitted fluorescence is similarly transmitted along the other half of the bundle and delivered to the emission spectrometer compartment of the instrument. The fibre-optic head is clamped in a retort stand and positioned 8 mm from the target surface.

The material of the fibre-optic cable is itself weakly fluorescent, emitting at around 310 nm, from short-wavelength ultraviolet (EEM not presented here) and so an excitation range of between 280 nm and 800 nm is used, with an increment of 10 nm. The emission is recorded across the same wavelength range as the liquid cuvette samples (200-900 nm), with a scan speed of 1000 nm/minute. As before, the excitation slit width is set to 8.0 nm, and the emission slit to 5.0 nm. The complete fluorescence data set for each solid sample is thus composed of 65,720 intensity measurements across excitation-emission space. For further details on the protocol used for fluorescence spectroscopy of both liquid and solid surface samples and the processing of EEMs, see [46–48].

Unlike fluorescence analysis of the bacterial liquid cultures, whereby the light-path through the cuvette serves to average across the population of cells present, the solid surfaces were analysed in specific locations. Figure 1 shows photographs of the solid surface samples typical of a hospital ward that were analysed here for background fluorescence. Where a sample was heterogeneous and composed of different materials, each of the components was analysed. For example, the nurse call button was analysed with the fibre-optic cable on the orange call button, the yellow light button, the white plastic casing, and the wire. Figure 1 indicates with white circles the different measurement locations for such heterogeneous samples. Homogenous material samples, such as the bed rail samples, were tested in a randomised location.

For both liquid cell cultures and solid surface samples, the EEMs were generated from the acquired datacube, and averaged between multiple samples by data transformation and analysis computer code, which was written by the lead author in Mathematica 7.0 (Wolfram Research, Champaign, USA). To smooth the recorded emission spectra and reduce the computational demands required for subsequent processing and plotting of the complete EEM, emission spectra were first averaged into bins 10 nm wide. The calculated EEM plots are presented on a log_10_ scale and displayed with rainbow color-coding and 15 contour lines. The appropriate range for color-coding of the fluorescence intensity was determined from an emission intensity histogram, as explained in [46]. No fluorescence signal was seen beyond the visible spectrum, and so in order to focus on the pertinent region, the results EEMs are not plotted beyond 700 nm.

## Results

### Bacterial cultures

**Figure 2:**
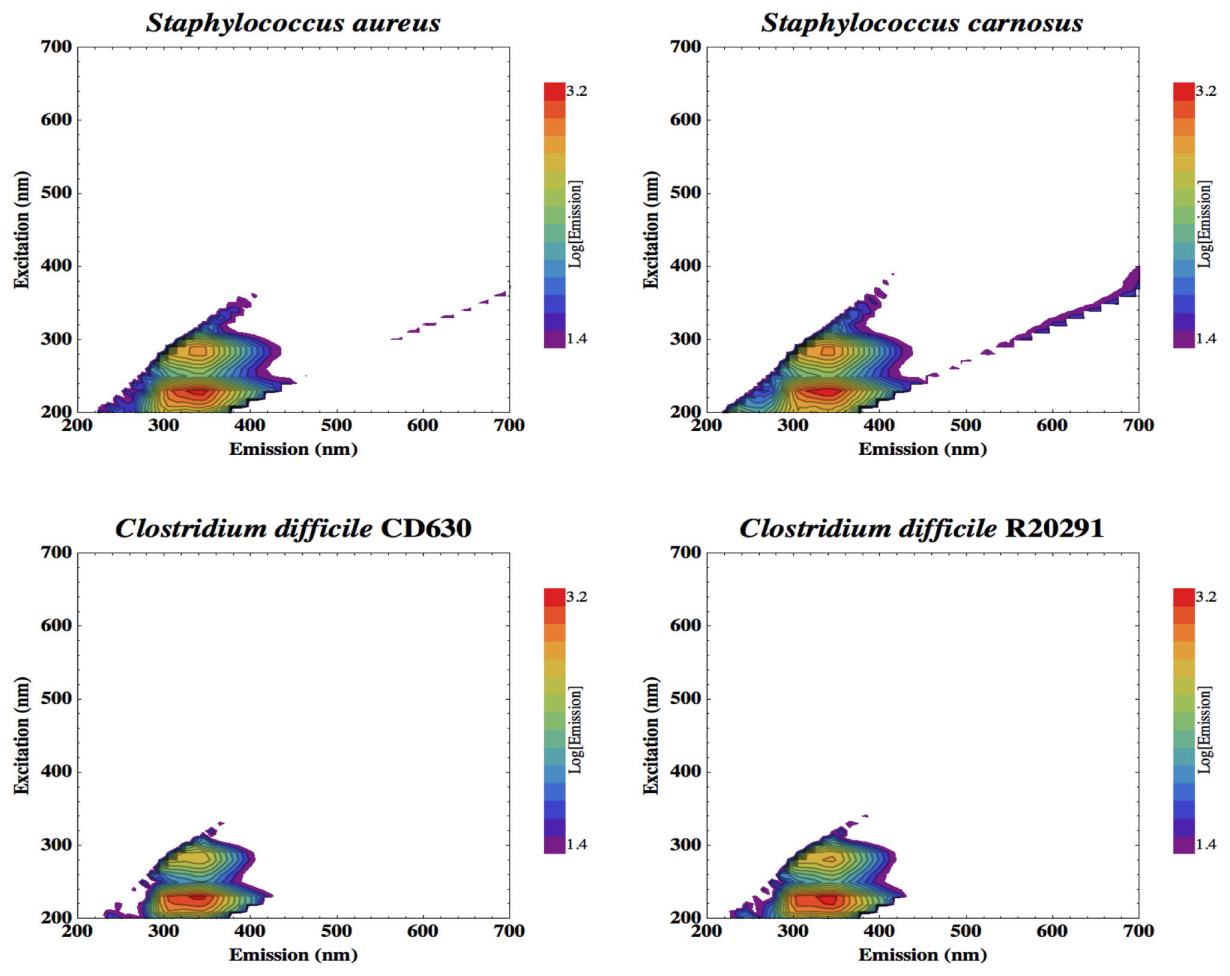
Excitation-emission matrices (EEMs) of the complete fluorescence response exhibited by clinically relevant Gram positive bacteria.

**Figure 3:**
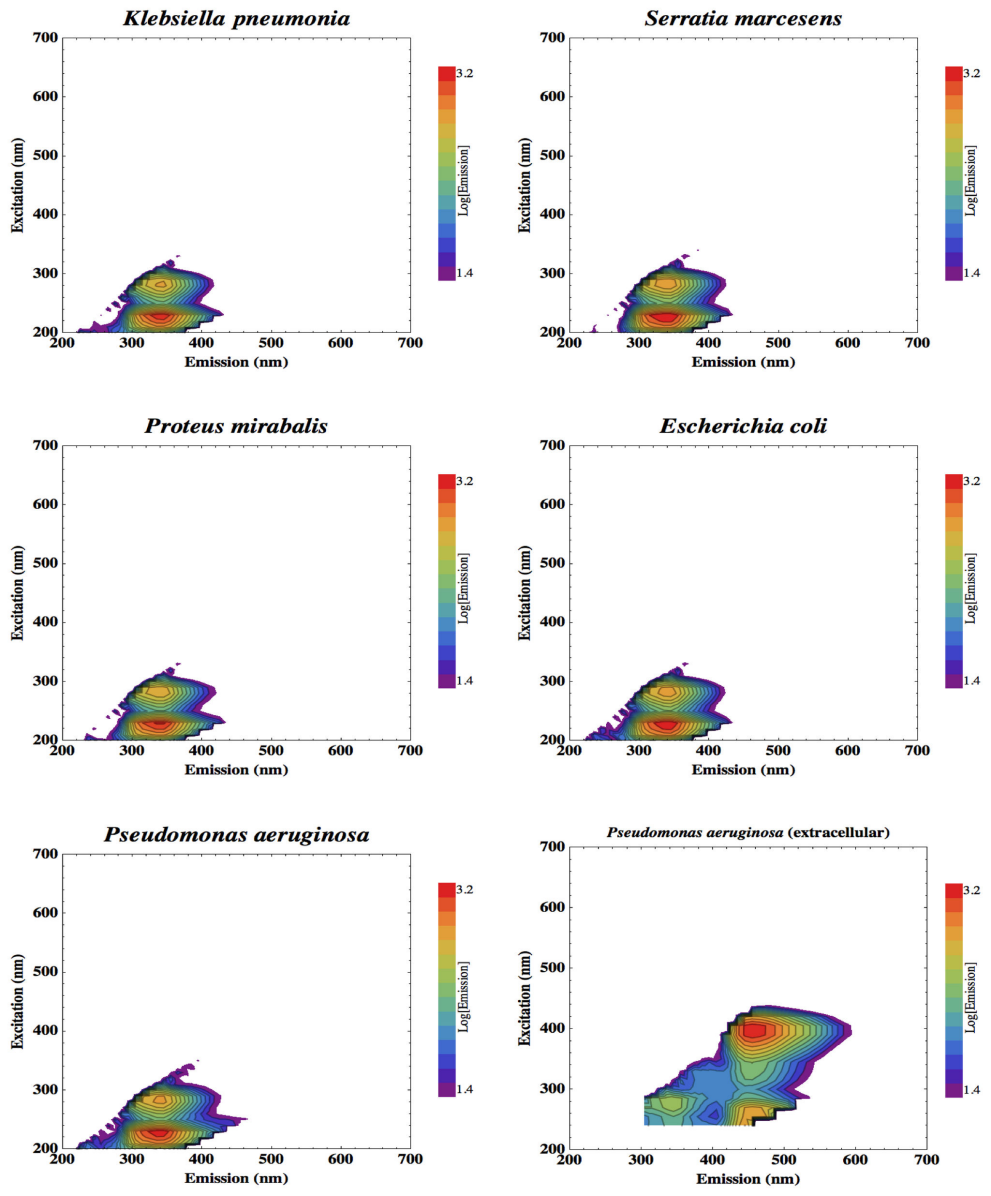
EEMs of the complete fluorescence response exhibited by clinically-important Gram negative bacteria. Data are also shown for *Pseudomonas aeruginosa* prepared so as to retain extracellular products such as the secreted siderophore, pyoverdine.

High-spectral-resolution EEMs, generated using over 85,000 excitation-emission pairings, were averaged from multiple samples of clinically-relevant bacteria. Figure 2 displays the EEMs produced for the four Gram-positive bacteria: *Staphylococcus aureus*, *Staphyloccus carnosus*, *Clostridium difficile* CD630, *Clostridium difficile* R20291. Figure 3 shows the five Gram-negative bacteria: *Klebsiella pneumoniae*, *Serratia marcesens*, *Proteus mirabalis*, *Escherichia coli*, and *Pseudomonas aeruginosa*. The EEM is also shown for the *Pseudomonas aeruginosa* minimal medium culture that was analyzed without the medium-washing step of the preparation protocol so as not to wash away the extracellular siderophore, pyoverdine.

### Typical hospital surfaces and materials

**Figure 4:**
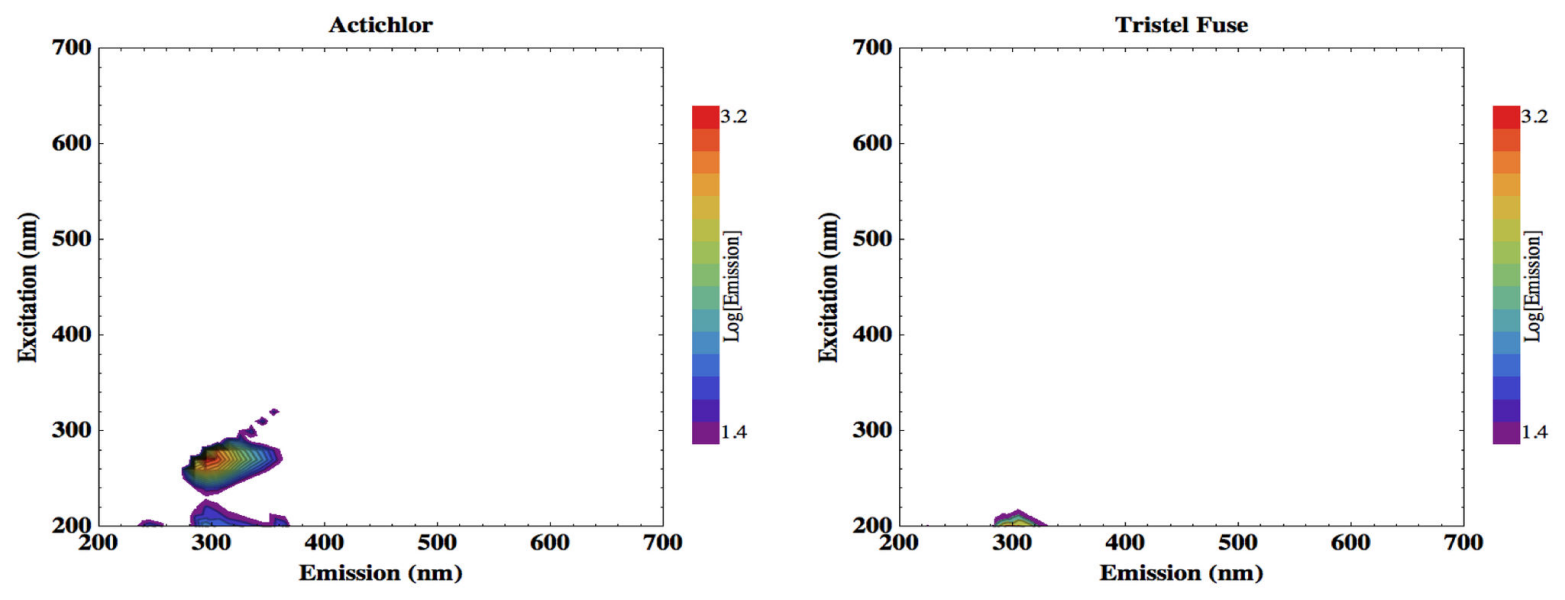
EEMs of two hospital cleaning fluids, Actichlor (left) and Tristel Fuse (right).

High-spectral-resolution EEMs were also generated of two biocidal cleaning products commonly used in hospitals: Actichlor and Tristel Fuse, as shown in Figure 4. Tristel Fuse can be seen to show no significant signal, but Actichlor does exhibit a fluorescent feature at an excitation-emission pairing of 280-300 nm

**Figure 5:**
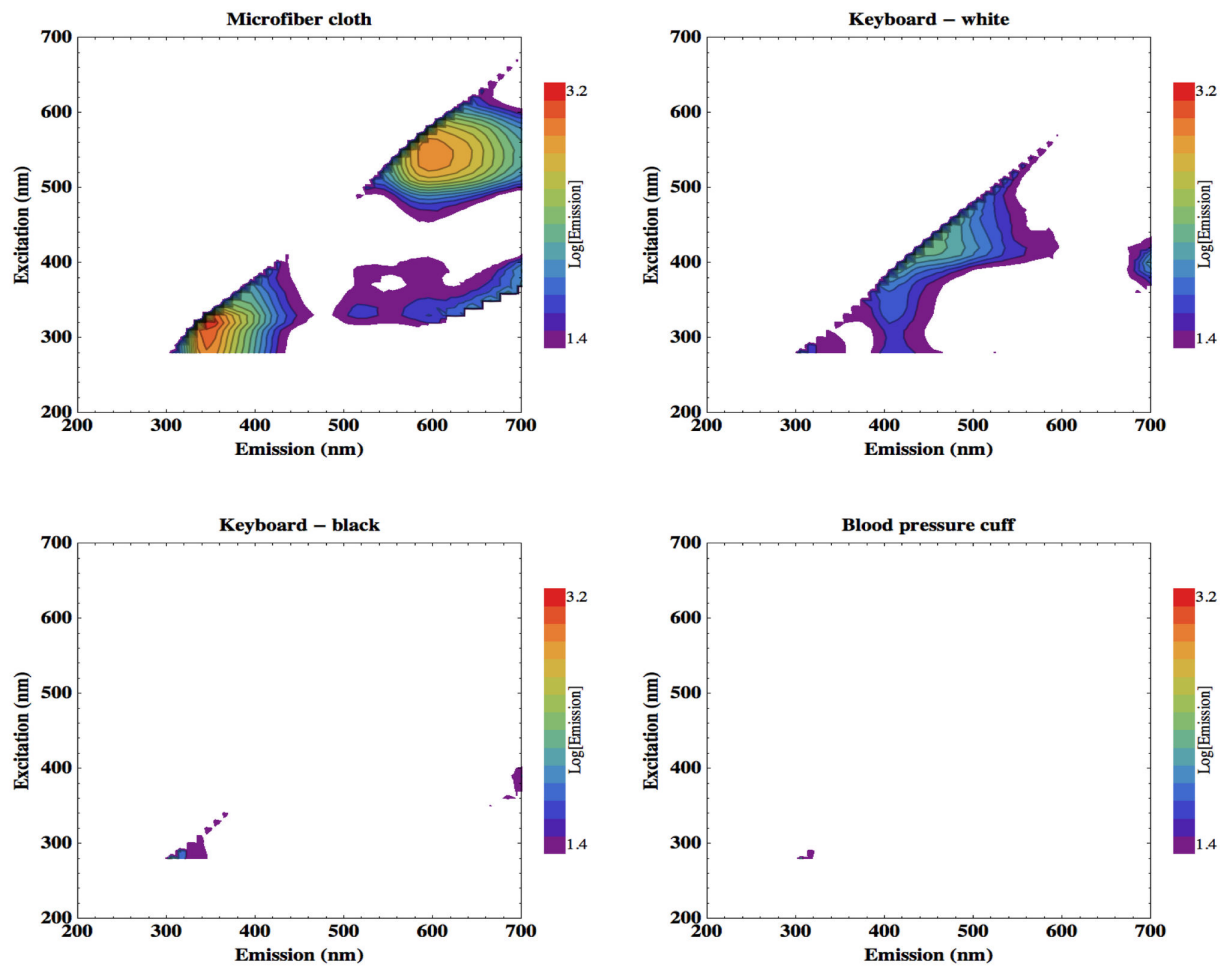
EEMs of the fluorescence response of equipment commonly found in a clinical setting. Microfibre cleaning cloth, two regions of clinical keyboard and a blood pressure cuff.

For the identification of possible sources of background fluorescence that may pose challenges for masking bacterial signal or present false-positive detections, multiple sampling sites on 10 different representative clinical surfaces were studied. Figure 5 shows the results acquired from the microfiber cleaning cloth, ward computer keyboard, and blood pressure cuff. The white regions of the computer keyboard cover exhibit slight fluorescence with emission around 440 nm. The microfiber cleaning cloth, however, exhibits more intense fluorescence, at two distinctive features. The first is elicited by short wavelengths, peak excitation around 300 nm, and has peak emission around 350 nm. The second is elicited by longer wavelength excitation light, around 545 nm, and re-emits around 600 nm.

**Figure 6:**
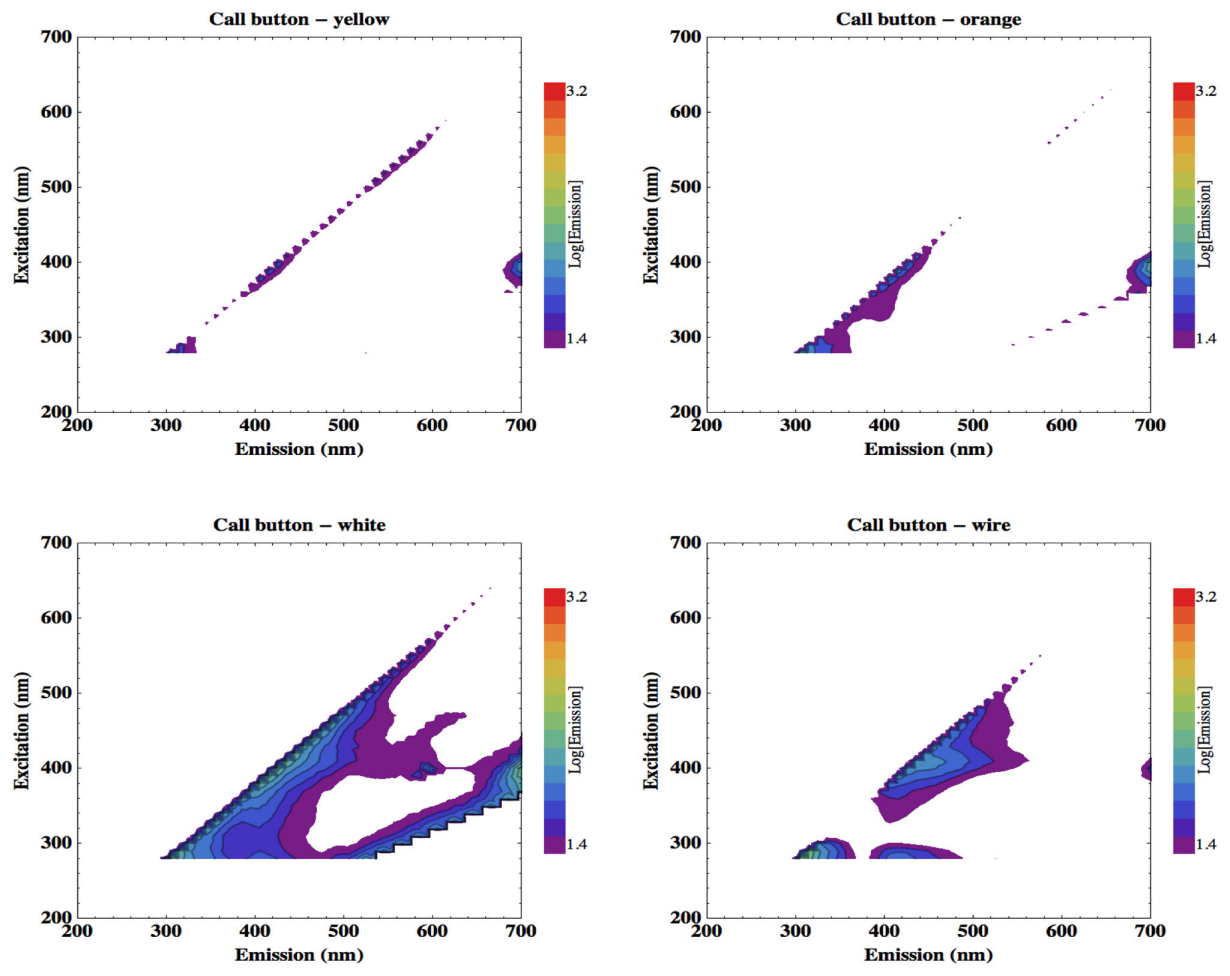
EEMs of the fluorescence response of different components of the patient nurse call button.

**Figure 7:**
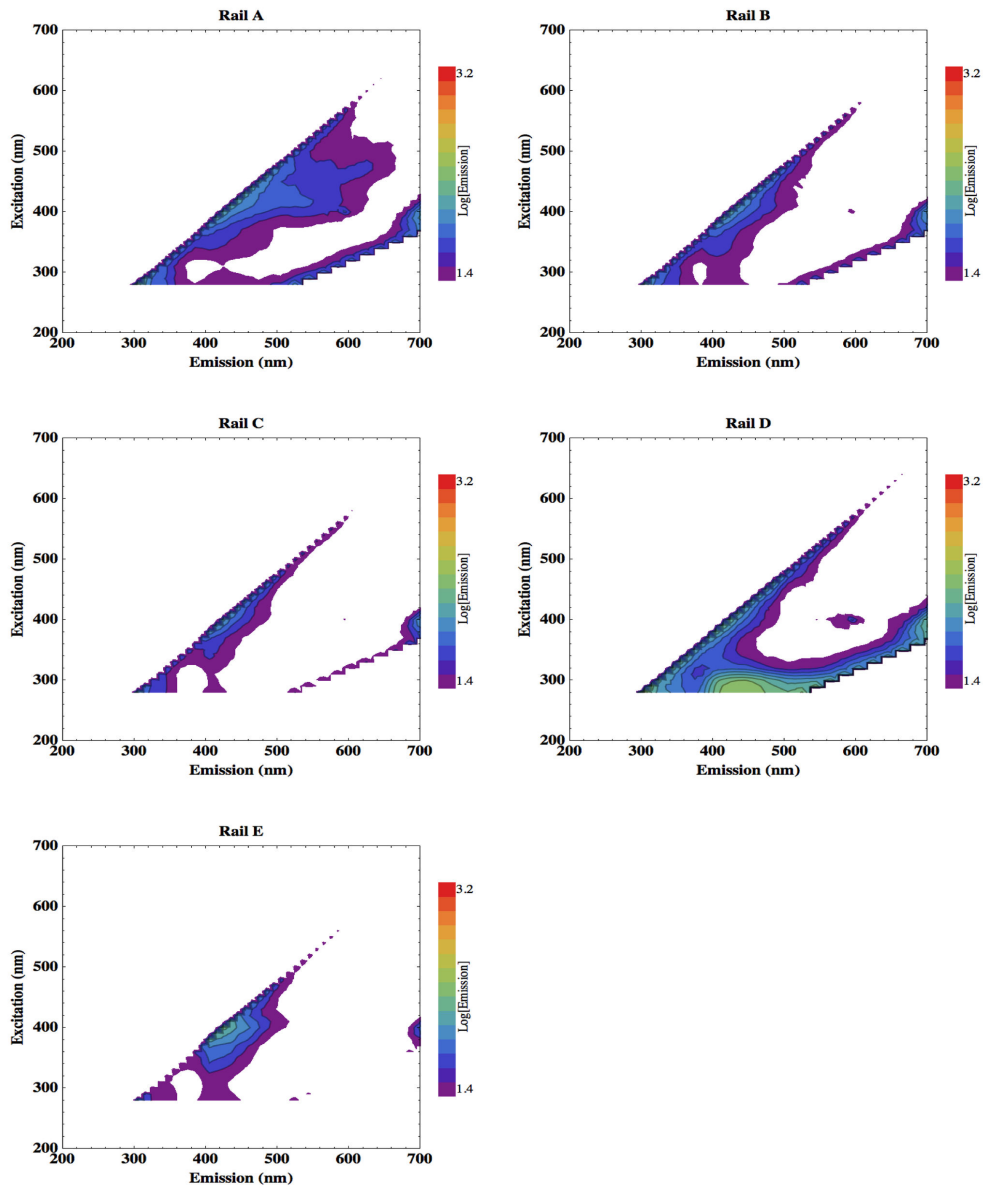
EEMs of different bed rail materials found on wards. Rail A: polypropylene with polyester powder coating; Rail B and Rail C: polypropylene; Rail D: nylon polyester painted mild steel; Rail E: stainless steel.

Figure 6 shows the EEMs acquired from the different component regions of the patient nurse call button, and Figure 7 displays those from the different handrail materials. Only rail sample D (nylon polyester painted mild steel) exhibits slight fluorescence emission between 400 and 500 nm.

## Discussion

The results are discussed in two parts. First, the analysis of HAI microorganisms, determining the complete fluorescence response and thus identifying the excitation and emission wavelengths of the most prominent fluorescence features. Second, the fluorescence observed in typical clinical surfaces or cleaning products to identify instances when background fluorescent noise may potentially mask the signal from contaminant cells.

Figures 2 and 3 show the EEMs of each of the bacteria cultured and analysed here. Each of these strains clearly shows a distinctive double-peak of tryptophan fluorescence. Tryptophan emission peaks at 340 nm, with two excitation maxima; at 230 nm and 280 nm. The tryptophan intensity is comparable between the strains studied. No other cellular fluorophores were detected here, except in the *Pseudomonas aeruginosa* culture prepared so as to retain extracellularly excreted compounds. Figure 3 shows the siderophore pyoverdine [11] to exhibit a broad fluorescent feature with emission between around 430 nm and 530 nm and peaking at 455 nm (blue), which is maximally excited at 395 nm. This fluorescence feature would also be easier to detect; Figure 3 shows pyoverdine emission to be four times the intensity of the tryptophan peak. Pyoverdine also emits in the visible band and so can be detected with standard optical components, not requiring UV sensitivity.

This fluorescence survey therefore reveals that the tryptophan peak would be the most reliable fluorophore for all cells to attempt to detect with a monitoring device for clinical contamination, with an additional target offered by the extracellular iron-scavenging compound released by *Pseudomonas*. Fluorescence extending through the 400-500 nm emission range, from excitation of around 350 nm, due to cellular metabolites such as NAD(P)H is also reported for many cells but is highly variable, dependent on the microbial strain and metabolic state and is not always detectable [44], and is much less intense than the tryptophan signal [46].

The next question, is what cleaning products and surfaces in a clinical setting might exhibit fluorescence in a similar spectral window to cellular tryptophan, and thus pose a potential problem of background fluorescence. Cleaning fluid residues or other materials that fluoresce at similar wavelengths to cellular fluorophores may contribute to false-positive detections in the operation of a contamination-monitoring device.

The Tristel Fuse solution shows no significant fluorescence, but Actichlor does exhibit a fluorescent feature at an excitation-emission pairing of 280-300 nm, which extends longwards into the spectral region occupied by the tryptophan signal. This measurement is, however, from a concentrated solution of the cleaning product in a liquid cuvette. A trace residue left after wiping clean a surface would yield a much lower background fluorescence. Furthermore, the fluorescence peak exhibited by Actichlor does not extend beyond 350 nm emission, whereas cellular fluorescence spreads to 400 nm, and so may be distinguished from potential masking by cleaning product residue by appropriate selection of filters on the detector. It is not anticipated, therefore, that trace residues of this particular cleaning product would pose a problem but this would need to be confirmed in a clinical setting.

Figure 7 shows that the plastic material of sample Rail D, nylon polyester painted onto mild steel, also shows a background fluorescence feature from excitation at 280 nm, emission peak at 440 nm, which is clear of the tryptophan peak. The EEM, however, does show signal at 340 nm emission which could mask tryptophan fluorescence.

Figure 5 (top left) shows that the microfibre cleaning cloth has two fluorescence features. The first of these, the short wavelength feature, appears at an ex-em pairing of around 300-350 nm. This is potentially problematic for a fluorescence-based bacterial detection device as cellular tryptophan has a peak ex-em pairing of 280-340 nm, and so may be masked by the fluorescence of microfiber cloths. This spectral overlap can be seen more clearly by comparing the EEMs produced by the microfiber cloth and a droplet of *Staphylococcus aureus* culture, the organism that when resistant to β-lactam antibiotics can cause MRSA outbreaks, dried onto a microscope slide to emulate clinical contamination. The sample had been concentrated tenfold from a mid-log growth culture by centrifuge, and had a population density of around 10^10^ cells/ml. Five microlitres of this sample were pipetted onto the slide to form a circular cell patch that was completely contained within the illumination spot of the spectrometer fibre-optic cable. Thus, the *S. aureus* surface sample contained a total of approximately 50,000 cells.

**Figure 8:**
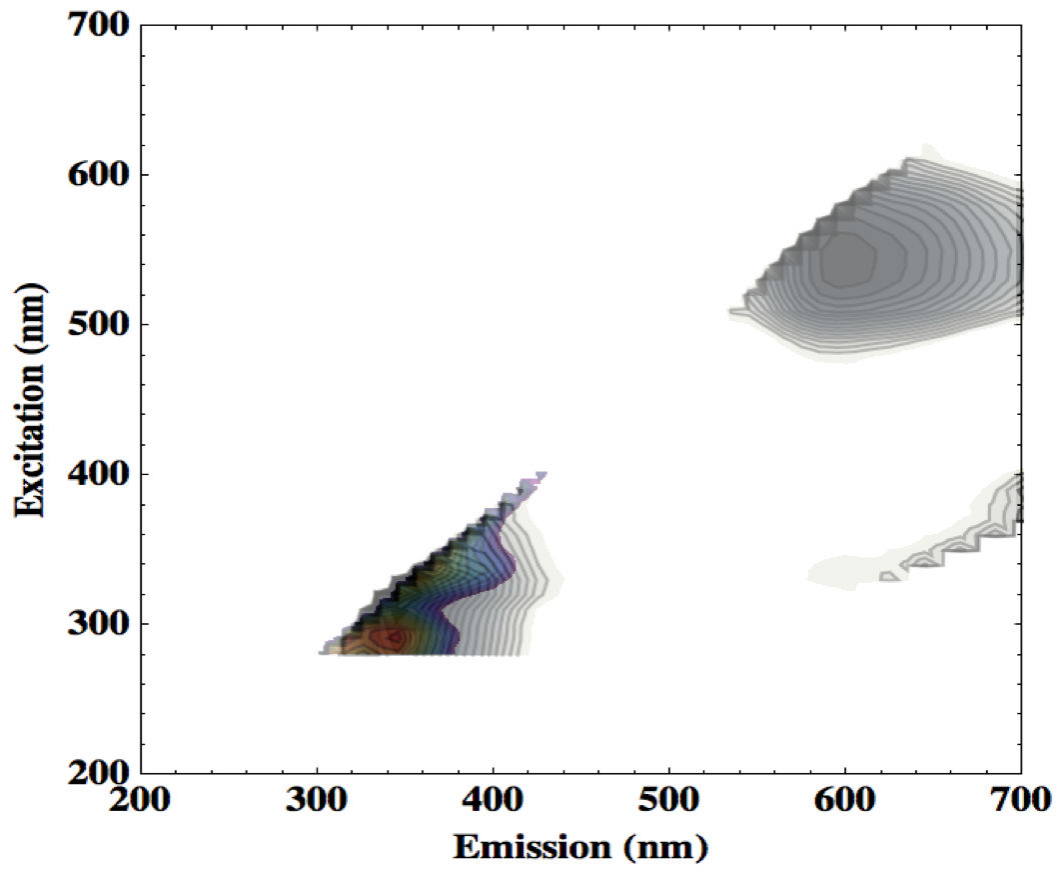
EEMs (arbitrary intensity units) produced by microfiber cleaning cloth (greyscale) and *S. aureus* (colour). Combined here so as to display the spectral overlap in the excitation-emission region occupied by tryptophan fluorescence.

Figure 8 displays the EEM produced by this dried droplet of *S. aureus* superimposed with the fluorescence response of the microfiber cloth to show the spectral similarity. The microfiber cloth is displayed in greyscale, and the *S. aureus* signal in colour.

Fluff residue or loose threads left on a clinical surface by the use of such a microfiber cloth may thus result in false positives for tryptophan detection, based on spectral similarity alone. A reliable strategy for excluding this form of false positive may be to involve machine vision algorithms in the imaging system of the detection device. A fibre of cleaning cloth, elongated and with distinct edges, would be readily distinguishable from an amorphous patch of bacteria-containing contamination on a clinical surface.

The additional fluorescent signature produced by extracellular pyoverdine from *Pseudomonas aeruginosa* (Figure 3; bottom-right), however, appears in a spectral window between the emission features from the microfiber cloth and so would allow detection of such contamination without potential background interference.

## Conclusions

All the examined bacterial strains exhibited a distinctive double-peak fluorescence feature of tryptophan, a protein-bound aromatic amino acid, but no other cellular fluorophore was detected. Thus, this fluorescence survey found that an emission peak of 340 nm is the cellular fluorescence signal to target for detection of bacterial contamination, maximally excited with a UV illumination source of 280 nm. Additionally, contaminating environmental reservoirs of *Pseudomonas aeruginosa* could be detected by the broad emission feature from pyoverdine. This intense fluorophore (a signal four times more intense that the tryptophan emission, and in the visible band) emits between about 430 and 530 nm, and is maximally excited at 395 nm. The cleaning fluid Tristel Fuse showed no significant fluorescence, however, Actichlor and the hand rail sample D (nylon polyester painted mild steel) do exhibit a fluorescence feature that partly coincides with the target tryptophan peak, and so may create a background signal on some surfaces that could mask that of contaminant bacteria. The majority of the materials analysed here, however, offer a spectral window through which bacterial contamination could be detected by fluorescence. A more intense fluorescence signal was observed with the microfiber cleaning cloth, which could pose a risk of false positives, but imaging would readily allow discrimination between a clearly-defined thread of cloth and an amorphous dried droplet of contaminated fluid.

Further work will focus on construction and testing a prototype device, building on these results, to illuminate with a UV source around 280 nm, shown here to maximally excite fluorescence of cellular tryptophan, and running trials to confirm successful detection of contaminant bacteria in a clinical setting rather than laboratory test conditions. The intended mode of operation of such a hand-held device will be in rapid-sweeping surveillance of surfaces.
